# lncRNA transcriptional initiation induces chromatin remodeling within a limited range in the fission yeast *fbp1* promoter

**DOI:** 10.1038/s41598-018-36049-0

**Published:** 2019-01-22

**Authors:** Satoshi Senmatsu, Ryuta Asada, Takuya Abe, Charles S. Hoffman, Kunihiro Ohta, Kouji Hirota

**Affiliations:** 1 Department of Chemistry, Graduate School of Science, Tokyo Metropolitan University, Minamiosawa 1-1, Hachioji-shi, Tokyo 192-0397 Japan; 2 Biology Department, Boston College, Chestnut Hill, MA 02467 USA; 3 Department of Life Sciences, The University of Tokyo, Meguro-ku, Tokyo 153-8902 Japan; 4 Universal Biology Institute, The University of Tokyo, Bunkyo-ku, Tokyo 113-0033 Japan

## Abstract

Long noncoding RNAs (lncRNAs) transcribed across gene promoters have been detected. These regulate transcription by mechanisms that have not been fully elucidated. We herein show that the chromatin configuration is altered into an accessible state within 290 bp downstream from the initiation site of metabolic-stress-induced lncRNAs (mlonRNAs) in the promoter of the fission yeast *fbp1* gene, whose transcription is massively induced upon glucose starvation. Chromatin upstream from *fbp1* is progressively altered into an open configuration, as a cascade of transcription of three overlapping mlonRNA species (-a, -b and -c in order) occurs with transcriptional initiation sites progressing 5′ to 3′ upstream of the *fbp1* promoter. Initiation of the shortest mlonRNA (mlonRNA-c) induces chromatin remodeling around a transcription factor-binding site and subsequent massive induction of *fbp1*. We identify the *cis*-element required for mlonRNA-c initiation, and by changing the distance between mlonRNA-initiation site and the transcription factor-binding site, we show that mlonRNA-initiation effectively induces chromatin remodeling in a limited distance within 290 bp. These results indicate that mlonRNAs are transcribed across the *fbp1* promoter as a short-range inducer for local chromatin alterations, and suggest that strict chromatin modulation is archived via stepwise mlonRNA-initiations.

## Introduction

Recent transcriptome analyses have revealed that most regions in the human genome are transcribed into RNAs, of which RNAs longer than 200 nucleotides possessing mRNA-like structure (carrying cap-structure and poly-A tail) without protein-coding potential are referred to as long noncoding RNA (lncRNA)^1^. Various functions of such RNAs have been identified in a range of biological processes^2,3^. lncRNAs transcribed within gene promoters play a role in the regulation of neighboring genes^3^. Several lncRNAs interact with polycomb repressive complex 2 (PRC2) and recruit it to target genes, leading to methylation of histone H3K27 following chromatin compaction^3,4^. Intergenic noncoding transcription at the budding yeast *SER3* gene promoter represses the expression of this gene^5^, and this repressive activity was observed even when >90% of the lncRNA sequence was replaced^6^. These data indicate that RNA polymerase II (RNAPII) transcription in the regulatory region is sufficient to mediate repression, and that lncRNA itself does not play a direct role. In *Schizosaccharomyces pombe* (fission yeast), upon glucose starvation, stepwise expression of lncRNAs at the *fbp1* gene promoter plays a critical role in chromatin modulation and subsequent gene activation^7^. This activation is mediated through two distinct mechanisms: (1) the lncRNA itself interacts with Tup1-like corepressors and thereby antagonizes the repressive function of the Tup1-like corepressors and facilitates binding of the Atf1 transcription factor^8^, and (2) RNAPII-mediated transcription of the lncRNAs mediate chromatin remodeling and further enhance Atf1 transcription-factor binding^8,9^.

**Figure 1:**
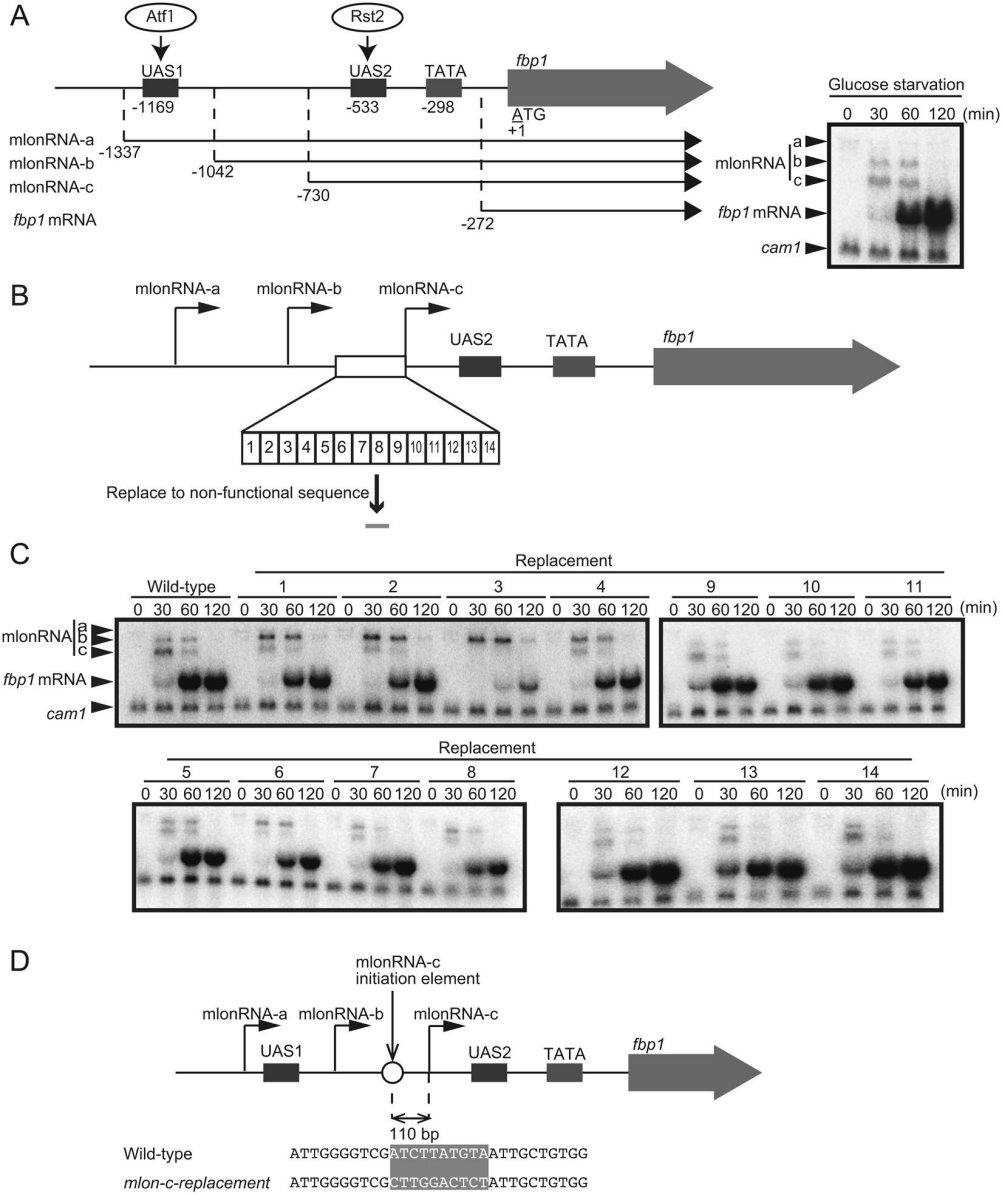
Identification of the *cis*-acting element required for mlonRNA-c transcriptional initiation. (**A**) Schematic drawing of the region upstream from *fbp1* containing upstream-activating sequences 1 and 2 (UAS 1 and UAS2), the binding sites for transcription factors Atf1 and Rst2, respectively. The mlonRNAs transcribed across the *fbp1* upstream region and *fbp1* mRNA are presented. The numbers indicate the transcription start-site of the *fbp1* transcripts and the distances of UAS1, UAS2, and the TATA box from the first ATG of *fbp1* ORF. Representative northern blot image showing expression of the mlonRNAs and *fbp1* mRNA during glucose starvation. Wild-type cells were grown to 2.0 × 10^7^ cells/mL in YER medium, then transferred to YED medium. Cells were harvested at the indicated times. *cam1* transcript was used as an internal control. (**B**) Schematic representation of segments covering the region upstream from the mlonRNA-c initiation site. We divided this region into 14 segments, then replaced each segment from the *act1* ORF. (**C**) Northern blot analysis to examine *fbp1* transcripts in cells carrying replacement sequences upstream from the mlonRNA-c initiation site. Cells were cultured and harvested as A. (**D**) The 10 nt sequence replaced in *mlon-c-replacement* cells is shaded.

Activation of the fission yeast *fbp1* gene as a result of glucose starvation stress is mediated by two transcription factors: Atf1 and Rst2^10,11^. Upon glucose starvation, these transcription factors bind to critical *cis*-acting binding sequences far upstream from the *fbp1* promoter (upstream-activating sequences 1 [UAS1] and 2 [UAS2])^10,12,13^ (Fig. 1A). In this upstream region, several species of lncRNA, referred to as metabolic stress-induced lncRNAs (mlonRNAs), are transcribed (Fig. 1A, mlonRNA-a, -b and -c in order). Initially, we had defined ‘mRNA type long ncRNA’ as ‘mlonRNA’, when the term ‘lncRNA’ had not been well recognized^14^. However, after this definition, the term ‘lncRNA’ has been used to mean the ‘mRNA-type long ncRNA’, and thus the definition of mlonRNA was changed to indicate ‘metabolic stress-induced lncRNAs’^15^. These mlonRNAs are transcribed in a stepwise manner from transcriptional initiation sites located in a 5′ to 3′ direction, leading to chromatin remodeling along their transcribed tract in the upstream region of *fbp1*^7,9^. RNAPII transcription of mlonRNAs passing across UAS2 is required for histone acetylation, disassembly, and subsequent Rst2 binding^7,9^. However, the mechanisms underlying the regulation of mlonRNA transcriptional initiation as well as the chromatin remodeling by mlonRNA transcription have not been elucidated. We herein identify the *cis*-element required for mlonRNA transcriptional initiation and demonstrate that mlonRNA transcription effectively induces chromatin remodeling within 290 bp downstream from the mlonRNA initiation site.

## Results

### Identification of the mlonRNA transcriptional-initiation element

During *fbp1* transcriptional activation upon glucose starvation, the chromatin state far upstream from the *fbp1* promoter is progressively altered into an open configuration. In this process, several species of mlonRNAs are transcribed in a stepwise manner with transcriptional initiation sites progressing in a 5′ to 3′ direction, thus inducing chromatin remodeling along the same tract^7,9^. However, how such stepwise mlonRNA transcriptions mediate chromatin remodeling is not well-understood. To investigate the significance of these stepwise mlonRNA transcriptions, we sought to selectively disrupt one of the transcripts in a cascade of transcription of three overlapping mlonRNAs. Since the initiation sites of mlonRNA–a and –b are close to essential *cis*-element, UAS1, and selective disruption of *cis*-elements for these mlonRNAs was difficult, we focused our efforts on disrupting the shortest mlonRNA-c transcription. To this end, we searched for the *cis*-element required for mlonRNA-c initiation by replacing 10–15 bp segments with *act1* ORF sequences in the region upstream from the mlonRNA-c transcription initiation site (Fig. 1B). mlonRNA-c transcription is completely lost by replacing a 10 bp segment at 110 bp upstream from the transcription initiation site (Fig. 1C, strain 3). We thus conclude that the *cis*-acting element required for mlonRNA-c initiation is present in this 10 nt sequence (5′-ATCTTATGTA-3′) (Fig. 1D). Moreover, cells in which this critical sequence had been replaced (*mlon-c-replacement* cells) exhibit a drastic reduction in *fbp1* mRNA transcription (Fig. 1C). These data are consistent with previously appreciated critical role of mlonRNAs in chromatin remodeling and subsequent transcription factor binding at UAS2^7,9^. Given the role played by mlonRNA initiation in chromatin opening, we hypothesized that there is a limitation in distance between the mlonRNA initiation site and the area of chromatin opening, such that the mlonRNA-a and -b initiation sites are too distant to promote chromatin opening at UAS2.

### Initiation of mlonRNA-b transcription 190 bp upstream from UAS2 bypasses the requirement for mlonRNA-c transcriptional initiation

**Figure 2:**
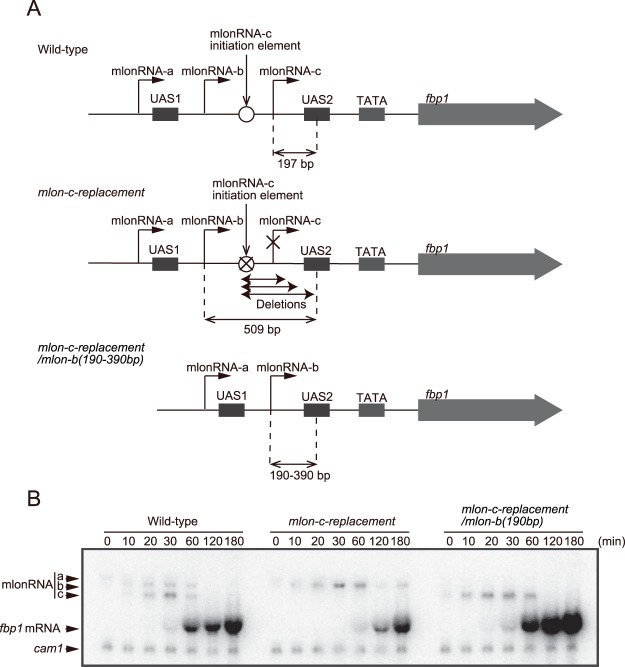
mlonRNA-b transcriptional initiation 190 bp upstream from UAS2 bypasses the requirement of mlonRNA-c transcriptional initiation for *fbp1* induction. (**A**) Schematic drawing of the region upstream from *fbp1* in wild-type, *mlon-c-replacement*, and *mlon-c-replacement/mlon-b(190–390* *bp)* cells. In *mlon-c-replacement/mlon-b(190–390* *bp)* cells, the distance between the mlonRNA-b initiation site and UAS2 was reduced to 190 bp, 240 bp, 290 bp, 340 bp, and 390 bp. (**B**) Northern blot analysis in wild-type, *mlon-c-replacement*, and *mlon-c-replacement/mlon-b(190* *bp)* cells. Cells were cultured and harvested as described in Fig. 1.

To determine whether mlonRNA initiation affects chromatin remodeling within a limited range, we deleted a 319 bp segment containing the mlonRNA-c initiation element, thus moving the mlonRNA-b initiation site closer to UAS2 (*mlon-c-replacement/mlon-b(190* *bp)*; Fig. 2A). In the *mlon-c-replacement* cells described in Fig. 1, the distance between the mlonRNA-b initiation site and UAS2 is 509 bp, whereas in the *mlon-c-replacement/mlon-b(190* *bp)* cells, the distance of the corresponding region is 190 bp, a distance similar to that of mlonRNA-c and UAS2 in wild-type cells (197 bp) (Fig. 2A). By placing the mlonRNA-b initiation site 190 bp upstream from UAS2, *fbp1* induction after glucose starvation is restored (Fig. 2B).

**Figure 3:**
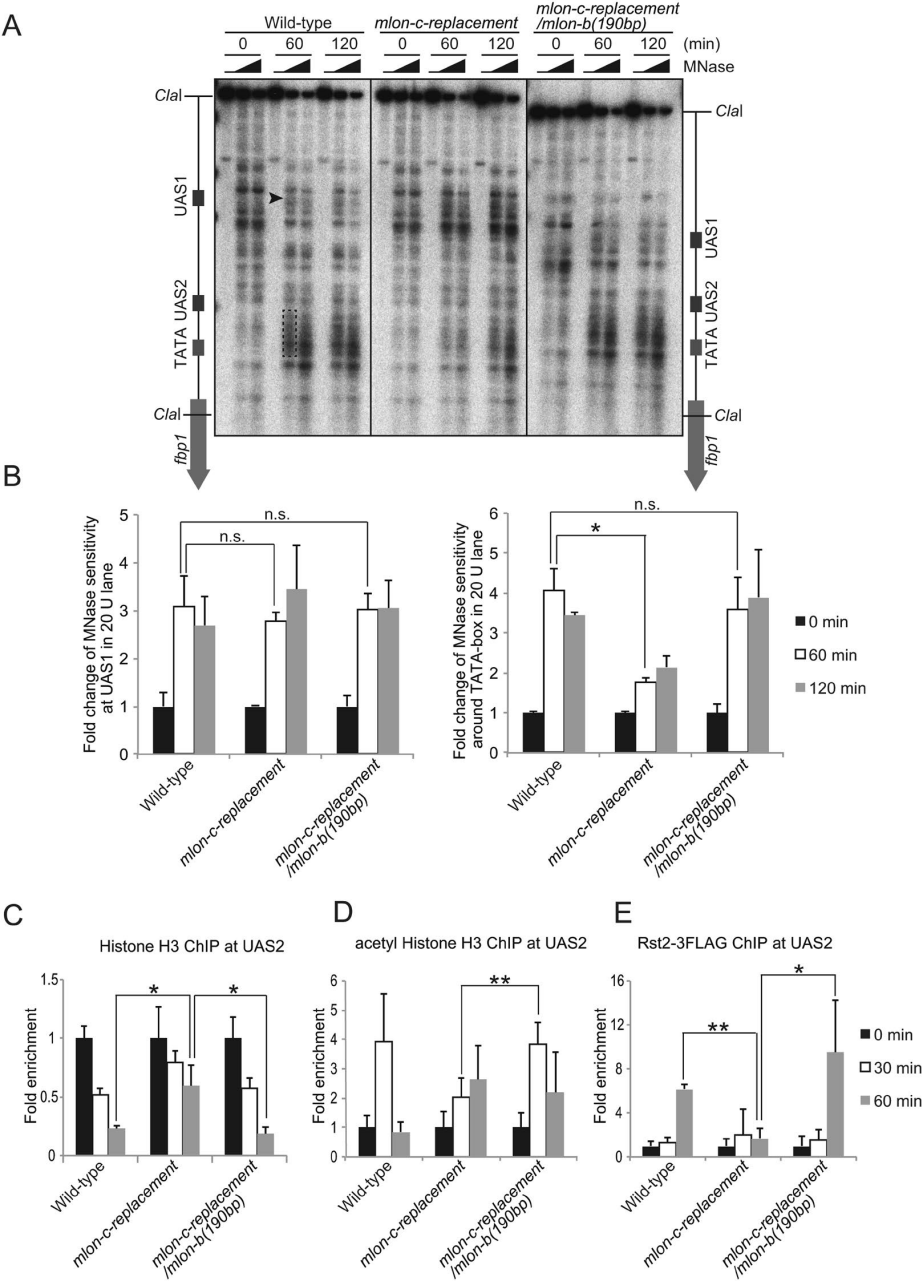
mlonRNA-b transcriptional initiation 190 bp upstream from UAS2 bypasses the requirement of mlonRNA-c transcriptional initiation for the efficient chromatin remodeling and transcription-factor binding. (**A**) Southern blot image showing MNase sensitive sites around the *fbp1* promoter in the indicated cells cultured in YED for the indicated time. The isolated chromatin was digested with 0, 20, or 50 units/ml of MNase at 37 °C for 5 min. Purified DNA was digested with *Cla*I and analyzed by Southern blotting. Black arrowhead indicates region with MNase-sensitive sites at UAS1. Dotted line indicates MNase-sensitive sites around UAS2-TATA box. (**B**) Histogram shows the quantification of MNase-sensitive bands in the UAS1 (Black arrowhead) and UAS2-TATA box region (Dotted line). The error bars show the standard deviation from at least two independent experiments. (C–E) ChIP analysis to examine histone binding (**C**), histone acetylation (**D**) and Rst2 binding (**E**) at UAS2 in wild-type, *mlon-c-replacement*, and *mlon-c-replacement/mlon-b(190* *bp)* cells. The relative increase in the ratio at the indicated time after glucose starvation is indicated. Error bars represent standard deviations from at least two independent experiments. *p*-value was calculated by a Student’s t-test: *p < 0.05, **p < 0.01 and n.s. (not significant, *p* > 0.05).

To determine if this recovery is indeed attributable to the restoration of chromatin remodeling at UAS2, we investigated the status of chromatin configuration. To this end, we employed an indirect end-labeling analysis involving partial digestion of chromatin DNA with micrococcal nuclease (MNase) to measure sensitivity to MNase reflecting open chromatin configuration. In wild-type cells, MNase sensitive sites appear near UAS1 followed by sites in the UAS2-TATA box region (Fig. 3A,B). In marked contrast, *mlon-c-replacement* cells exhibit less prominent MNase sensitive region around UAS2-TATA box (Fig. 3A,B). However, by moving the mlonRNA-b initiation site closer to UAS2 (*mlon-c-replacement/mlon-b(190* *bp)*), MNase sensitivity around UAS2-TATA box region is completely restored to wild-type level (Fig. 3A,B). Consistently, histone binding at UAS2 is progressively reduced in wild-type cells, diminishing to less than 30% of the original state at 60 min after glucose starvation, whereas the *mlon-c-replacement* cells exhibit impaired histone disassembly, with approximately 60% of histones remaining at 60 min (Fig. 3C). This histone-disassembly defect is also completely restored by moving the mlonRNA-b initiation site closer to UAS2 (*mlon-c-replacement/mlon-b(190* *bp)*) (Fig. 3C).

We further analyzed the kinetics of histone acetylation and transcription-factor binding at UAS2. At 30 min after glucose starvation, histone acetylation is transiently induced over three fold over the original state in wild-type cells, while *mlon-c-replacement* cells exhibit delayed and reduced histone acetylation in this region (Fig. 3D). Moreover, Rst2 binding at UAS2 increases > five-fold in the wild-type cells, while the *mlon-c-replacement* cells exhibit little Rst2 binding after glucose starvation (Fig. 3E). These defects of histone acetylation and Rst2 binding observed in the *mlon-c-replacement* cells are significantly rescued in *mlon-c-replacement/mlon-b(190* *bp)* cells (*p* < 0.01 and *p* < 0.05, respectively) and this cell line shows very similar kinetics to those of the wild-type cells (Fig. 3D,E). These results demonstrate that mlonRNA-b initiation 190 bp upstream from UAS2 efficiently promotes chromatin remodeling at UAS2, and bypasses the requirement of mlonRNA-c transcriptional initiation for *fbp1* mRNA induction. We conclude that mlonRNA transcriptional initiation induces chromatin remodeling within a limited distance from the initiation site.

### The effective range of mlonRNA transcription-induced chromatin remodeling is approximately 290 bp

**Figure 4:**
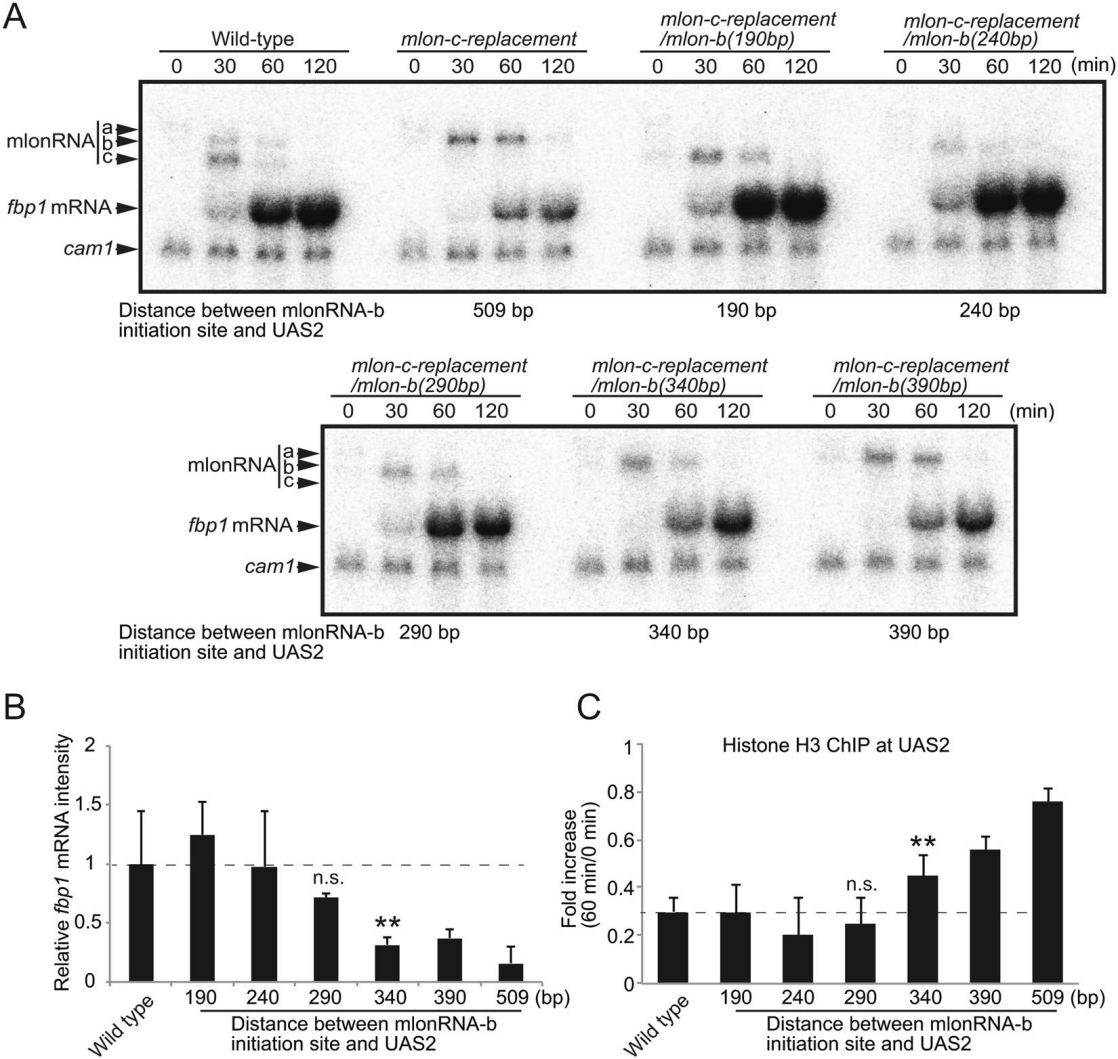
mlonRNA transcriptional initiation promotes chromatin remodeling within an effective range of approximately 290 bp. (**A**) Northern blot analysis in wild-type, *mlon-c-replacement*, and *mlon-c-replacement/mlon-b(190–390* *bp)* cells. Cells were cultured and harvested as described in Fig. 1. (**B**) *fbp1* mRNA transcripts were quantified using Image J (https://imagej.nih.gov/ij/) and intensities at 60 min after initiation of glucose starvation relative to those of wild-type cells are presented. Error bars represent standard deviation from three independent experiments (**C**) Histone binding 60 min after initiation of glucose starvation relative to 0 min (glucose rich condition) was assessed by ChIP analysis. Error bars show the standard deviation from at least two independent experiments. *p*-value was calculated by a Student’s t-test: *p < 0.05, **p < 0.01 and n.s. (not significant, *p* > 0.05).

We next sought to identify the limitation in the distance from mlonRNA initiation to induce chromatin remodeling at UAS2. To this end, we examined various distances (240, 290, 340, and 390 bp) between the mlonRNA-b initiation site and UAS2 by generating *mlon-c-replacement/mlon-b(240* *bp)*, *mlon-c-replacement/mlon-b(290* *bp)*, *mlon-c-replacement/mlon-b(340* *bp)*, and *mlon-c-replacement/mlon-b(390* *bp)* cells (Fig. 2A). We found that the induction of *fbp1* mRNA lessens as the distance between the mlonRNA-b initiation site and UAS2 increases (Fig. 4A,B). The *mlon-c-replacement/mlon-b(190–290* *bp)* cells exhibit higher or wild-type levels of *fbp1* mRNA induction, whereas the *mlon-c-replacement/mlon-b(340–509* *bp)* cells show significantly lower levels of induction compared to wild-type cells (Fig. 4A,B, *p* < 0.01). The higher level of *fbp1* expression in the *mlon-c-replacement/mlon-b(190* *bp)* strain might be attributable to the alteration of genome geometry in this region by sequence deletion, since *fbp1* activation is also affected by the local DNA-loop that brings UAS1 and UAS2 into close spatial proximity^16^. We next evaluated histone disassembly at 60 min after glucose starvation in the cell lines with various segment lengths between the mlonRNA-b initiation site and UAS2. In the wild-type cells, histone H3 binding is reduced to below 30% of the original state. In the *mlon-c-replacement* cells with 190 bp, 240 bp, and 290 bp segments between the mlonRNA-b initiation site and UAS2, histone disassembly is very similar to that of the wild-type(Fig. 4C, n.s., *p* > 0.05). In marked contrast, the *mlon-c-replacement* cells with the 340, 390, and 509 bp segments exhibit histone binding levels of 50%, 60%, and 75%, respectively (Fig. 4C, *p* < 0.01). These data suggest that mlonRNA initiation efficiently induces histone disassembly within a limited distance of ~290 bp from the initiation site, while chromatin remodeling gradually decreases as the distance from the mlonRNA initiation site increases beyond 290 bp.

## Discussion

Transcriptional activation of the fission yeast *fbp1* gene is preceded by RNAPII-mediated transcription of a series of lncRNAs, referred to as mlonRNAs, in a stepwise manner. Transcription of the mlonRNAs induces chromatin remodeling in their transcribed regions, thereby contributing to the access of transcription factors to the *fbp1* regulatory elements^7^. In this study, we identified the *cis*-acting sequence required for transcriptional initiation of the mlonRNA-c transcript, which is the transcript nearest to the *fbp1* promoter. Loss of mlonRNA-c transcription due to inactivating this sequence resulted in a critical defect in chromatin remodeling at the Rst2 transcription-factor binding site located downstream from the mlonRNA-c initiation site. These results indicate the pivotal role played by mlonRNA transcriptional initiation in chromatin remodeling, as the mlonRNA-b transcript overlaps with the mlonRNA-c transcript, showing that transcriptional elongation through this region is not sufficient to promote chromatin remodeling. An interesting possibility is that the RNAPII complex that transcribes mlonRNA might be distinct from the usual RNAPII complex associated with the TATA box, and such mlonRNA transcribing RNAPII may play a role in inducing chromatin remodeling. Should this be true, this mlonRNA-RNAPII would function locally, around the initiation site, since RNAPII accumulates around its transcription-start site and dissociates from the initiation complex after promoter clearance^17^. This is indeed the case, as we found that mlonRNA initiation efficiently induces chromatin remodeling only within 290 bp of the initiation site (Fig. 4). This range is comparable to the length of two adjacent nucleosomes (294 bp). This is intriguing, since two neighboring nucleosomes tend to be modified in a similar manner^18,19^. Our current observations further support the notion raised by previous studies that non-canonical transcripts can regulate gene expression^20,21^, and provides further evidence that lncRNA-transcribing RNAPIIs can act as “pioneers” to rearrange the chromatin array^22^.

In the analysis to identify the limitation in the distance from mlonRNA initiation to induce chromatin remodeling, we found slightly higher level of *fbp1* expression in the *mlon-c-replacement/mlon-b(190* *bp)* strain (Fig. 4B). This higher expression may be attributable to the alteration of the distance between UAS1 and UAS2 by sequence deletion, since *fbp1* activation is also affected by the local DNA-loop that brings UAS1 and UAS2 into close spatial proximity^16^. It is possible that such local DNA-loop structure might be also affected by the mlonRNAs initiations. Since chromatin remodeling is prerequisite for the DNA-loop formation in glucose starvation^16^, chromatin remodeling defects caused by the mlonRNA-c replacement may indirectly affect on the local DNA-loop structure. Important questions for future studies concern a possible direct contribution of mlonRNA initiation to the formation of such local DNA-loop structure.

We note that the positional relationships between each of the mlonRNA initiation sites and their target elements are very similar, as evidenced by the fact that the distances between (1) the mlonRNA-b initiation site and the *cis*-element required for mlonRNA-c transcription and (2) the mlonRNA-c initiation site and the *cis*-element (UAS2) required for *fbp1* mRNA are 192 bp and 197 bp, respectively. Stepwise transcriptional activation of several species of mlonRNA may be responsible for converting chromatin into an open configuration upstream from *fbp1*, thereby contributing to the strict binding-order regulation of transcription factors. The transcription factor Rst2 binding is mediated by a local DNA-loop that brings UAS1 and UAS2 into close spatial proximity^16^. The formation of an open chromatin configuration upstream from *fbp1* is a prerequisite for the formation of this higher-order structure. Thus, the strict regulation of chromatin configuration by a cascade of mlonRNA transcriptions and the subsequent formation of a local DNA-loop structure should cooperatively control *fbp1* transcription. Limiting the range in which mlonRNA can effectively induce chromatin remodeling may contribute to this strict chromatin modulation by prohibiting improper chromatin opening. This stepwise regulation via the cascade of mlonRNA transcriptions within the range limitation for each mlonRNA initiation may mediate this strict modulation of gene expression and the appropriate response to glucose starvation stress^23^.

In the *S. pombe* core environmental stress response (CESR) gene, the first nucleosome downstream from the transcription-start site (+1 nucleosome) is removed during gene activation. This process is mediated in a histone H3 acetyltransferase, Gcn5 dependent manner^24^. Gcn5 is recruited through Atf1 binding at UAS1 in the *fbp1* gene and is involved in the activation of this gene^8,9^. It is thus possible that chromatin eviction at UAS2 through mlonRNA-c initiation is mediated by a process involving Atf1 and Gcn5. This hypothesis is supported by the fact that expression of mlonRNA-c and subsequent chromatin remodeling is missing in *atf1*^−^ cells^7^. Interestingly, loss of the Tup1-like corepressors in *atf1*^−^ cells totally rescues the defect in *fbp1* induction without recovering mlonRNA-c initiation^7^. These data suggest a possible counteractive regulation between mlonRNA-initiation-mediated chromatin opening and Tup1-like-corepressor-mediated chromatin compaction. Since mutant fission yeast cells lacking Tup1-like corepressors show inappropriate, nonspecific *fbp1* transcription^25^, this counteractive regulation might be pivotal for precise *fbp1* activation resulting from environment stresses.

## Methods

### Fission-yeast strains, genetic methodology, and cell culture

The fission yeast strains used in this study are listed in Supplementary Table 1. YER medium (yeast extract containing 6% glucose) and YED medium (yeast extract containing 0.1% glucose and 3% glycerol) were used for glucose repression and starvation, respectively^26^. Transformation was performed using the lithium-acetate method as previously described^27^.

### Primers

The primer sequences used in this study are listed in Supplementary Table S2.

### Construction of strains with sequence replacements upstream from the mlonRNA-c initiation site

The *fbp1* promoter region including the mlonRNA-c upstream sequence was amplified using a primer set (p1 and p2) and cloned in pCR^®^-Blunt II-TOPO^®^ (Invitrogen). Replacement of the region upstream from mlonRNA-c with *act1* ORF was performed by PCR using the primer sets described in Supplemental Table 2. For replacement numbers 1–14, primer pairs p1 and p3-16, p2 and p17-30 were used respectively. The resulting fragments were purified using QIAquick gel extraction kit (Qiagen). Pairs of fragments were used as templates for PCR amplification using the primer set p1 and p2 to generate replacement constructs for numbers 1–14. Fission yeast cells carrying the *ura4* marker gene in the *fbp1* promoter were transformed using plasmid carrying the *fbp1* upstream sequence, in which a part of the segment upstream from the mlonRNA-c initiation site was replaced with the *act1* ORF sequence. To isolate the *fbp1* upstream-sequence replacement cells, transformants were selected for uracil auxotrophy using SD plates containing 5-FOA and uracil.

### Construction of *mlon-c-replacement* cells with sequence deletions between the mlonRNA-c initiation element and UAS2

To construct *mlon-c-replacement* cells with various deletion lengths in the region between the mlonRNA-c initiation element and UAS2, we deleted regions -937 to -619, -669, -719, -769, and -819 (relative to the first A in the ORF as +1) by PCR, using the primer sets p31 and p32 to p36 as described in Supplemental Table 2. We used the *fbp1* upstream region cloned in pCR^®^-Blunt II-TOPO^®^ (Invitrogen) as a template. PCR products were phosphorylated by T4 Polynucleotide Kinase (Takara Bio) and ligated using T4 DNA Ligase (TOYOBO). Fission yeast cells carrying the *ura4* marker gene in the *fbp1* promoter were transformed with these plasmid constructs and selected for uracil auxotrophy, as above.

### Construction of *rst2-3flag* strains

All *rst2-3flag* strains were constructed as described previously^28^.

### Northern blot, chromatin immunoprecipitation, and micrococcal nuclease (MNase) digestion assay

Northern blotting and chromatin immunoprecipitation (ChIP) were performed as described previously^29^. DNA probes for *fbp1* and *cam1* were amplified by PCR using primer sets p37/p38 and p39/p40, respectively. Anti-histone H3 (Abcam), anti-acetyl histone H3 (millipore), and anti- DYKDDDDK antibody (Wako) were used for the ChIP analysis. DNA concentrations were quantified using Thermal Cycler Dice Real Time (Takara Bio) and THUNDERBIRD^®^ SYBR qPCR Mix (TOYOBO). Primer sets p41/p42 at UAS2 and p43/p44 at the *prp3* locus were used for quantitative PCR analysis. MNase digestion assays were performed as described previously^16^.

## Electronic supplementary material

Supplementary Information
