# Supplementary material for: lncRNA transcriptional initiation induces chromatin remodeling within a limited range in the fission yeast fbp1 promoter
Source: Sci Rep. 2019 Jan 22;9:299. doi: 10.1038/s41598-018-36049-0 (PMC6342983; doi:10.1038/s41598-018-36049-0)
Supplement: Supplementary file 1 — Supplementary Information [file 41598_2018_36049_MOESM1_ESM.pdf]

lncRNA transcriptional initiation induces chromatin remodeling within a limited range in the fission yeast *fbp1* promoter

Satoshi Senmatsu[1*], Ryuta Asada[1*], Takuya Abe[1], Charles S. Hoffman[2], Kunihiro Ohta[3,4] and Kouji Hirota[1,†]

[1]Department of Chemistry, Graduate School of Science, Tokyo Metropolitan University, Minamiosawa 1-1, Hachioji-shi, Tokyo, 192-0397, Japan

[2]Biology Department, Boston College, Chestnut Hill, MA 02467, USA.

[3]Department of Life Sciences, The University of Tokyo, Meguro-ku, Tokyo 153-8902, Japan

[4]Universal Biology Institute, The University of Tokyo, Bunkyo-ku, Tokyo 113-0033, Japan

[*]These authors equally contributed to this work.

[†]Correspondence:Kouji Hirota Ph.D. (khirota@tmu.ac.jp)

Department of Chemistry, Graduate School of Science, Tokyo Metropolitan University, Minamiosawa 1-1, Hachioji-shi, Tokyo, 192-0397, Japan Tel:+81-42-677-2542, Fax: +81-42-677-2542

Supplementary Information: Table S1-S2, and Figure S1-S5

Supplementary Table S1 Fission yeast strains used in this study

Strain	Genotype
SPH2	h^+ *ura4-D18*
SPH20	h^- *ade6-M26 rst2-3flag<<kanMX6 leu1-32*
SPH280	h^+ *ura4-D18 mlon-c promoter(-871 to -862)::act1(+436 to +445)*
SPH281	h^+ *ura4-D18 mlon-c promoter(-861 to -852)::act1(+446 to +455)*
SPH282	h^+ *ura4-D18 mlon-c promoter(-851 to -842)::act1(+456 to +465)*
SPH283	h^+ *ura4-D18 mlon-c promoter(-841 to -827)::act1(+466 to +480)*
SPH323	h^+ *ura4-D18 mlon-c promoter(-826 to -817)::act1(+456 to +465)*
SPH324	h^+ *ura4-D18 mlon-c promoter(-816 to -807)::act1(+456 to +465)*
SPH350	h^+ *ura4-D18 mlon-c promoter(-806 to -797)::act1(+456 to +465)*
SPH351	h^+ *ura4-D18 mlon-c promoter(-796 to -787)::act1(+456 to +465)*
SPH352	h^+ *ura4-D18 mlon-c promoter(-786 to -777)::act1(+456 to +465)*
SPH353	h^+ *ura4-D18 mlon-c promoter(-776 to -767)::act1(+456 to +465)*
SPH354	h^+ *ura4-D18 mlon-c promoter(-766 to -757)::act1(+456 to +465)*
SPH355	h^+ *ura4-D18 mlon-c promoter(-756 to -747)::act1(+456 to +465)*
SPH356	h^+ *ura4-D18 mlon-c promoter((-746 to -737)::act1(+456 to +465)*
SPH357	h^+ *ura4-D18 mlon-c promoter(-736 to -727)::act1(+456 to +465)*
SPH406	h^+ *ura4-D18 mlon-c promoter(-937 to -619)*Δ
SPH420	h^+ *ura4-D18 mlon-c promoter(-851 to -842)::act1(+456 to +465) rst2-3flag<<kanMX6*
SPH421	h^+ *ura4-D18 mlon-c promoter(-937 to -619)*Δ *rst2-3flag<<kanMX6*
SPH436	h^+ *ura4-D18 mlon-c promoter(-937 to* -669*)*Δ
SPH437	h^+ *ura4-D18 mlon-c promoter(-937 to* -719*)*Δ
SPH438	h^+ *ura4-D18 mlon-c promoter(-937 to* -769*)*Δ
SPH439	h^+ *ura4-D18 mlon-c promoter(-937 to* -819*)*Δ

Supplementary Table S2 Primers used in this study

Primer	Sequence
p1	CATTGACTCCGATATCTCGG
p2	GCACCATACATAGTATAGCC
p3	TGGTACGACCTTGATGTCTAGCAATTGTGTATG
p4	ACGATACCAGTGCCTATGATTTGATGTCTAGC
p5	AGAGTCCAAGCGACCCCAATTGCCTATG
p6	GGTAACACCATCACCTACATAAGATCGACCCCAATTG
p7	AGAGTCCAAGCATACCCACAGCAATTACATAAG
p8	AGAGTCCAAGACCTCAATTGCATACCCAC
p9	AGAGTCCAAGTTCATCACCGACCTCAATTG
p10	AGAGTCCAAGGACCGCTGCTTTCATCAC
p11	AGAGTCCAAGAGGCGAGCATGACCGCTG
p12	AGAGTCCAAGAAGGATTGAGAGGCGAG
p13	AGAGTCCAAGTACATTCGTAAAGGATTGAGAGG
p14	AGAGTCCAAGACTCCCTTAATACATTCGTAAAG
p15	AGAGTCCAAGATGGCATGGCACTCCCTTAATAC
p16	AGAGTCCAAGATGGCATGGCATGGCATG
p17	GGTCGTACCAATTGGGGTCGATCTTATGTAATTG
p18	CTGGTATCGTATCTTATGTAATTGCTGTGGG
p19	CTTGGACTCTATTGCTGTGGGTATGCAATTG
p20	GGTGATGGTGTTACCCAATTGAGGTCGGTGATG
p21	CTTGGACTCTCGGTGATGAAAGCAGCGG
p22	CTTGGACTCTAGCAGCGGTCATGCTCG
p23	CTTGGACTCTATGCTCGCCTCTCAATCC
p24	CTTGGACTCTCTCAATCCTTTACGAATGTATTAAG
p25	CTTGGACTCTTACGAATGTATTAAGGGAGTGC
p26	CTTGGACTCTTTAAGGGAGTGCCATGC
p27	CTTGGACTCTGCCATGCCATGCCATGC
p28	CTTGGACTCTGCCATGCCATTTTCTCAGTC
p29	CTTGGACTCTTTTCTCAGTCACGTAAACCTC

p30	CTTGGACTCTACGTAAACCTCGATACGATAC
p31	AACTTGAACTTGTTTTCTCGTGC
p32	GTTTGGTAAGCCGGCTTC
p33	CAGTATTATAGGAAAGATGAATAGGGTG
p34	CCTCGATACGATACAAGCTCTAAC
p35	GTATTAAGGGAGTGCCATGCC
P36	GGTCGGTGATGAAAGCAGC
p37	CGCCGATACAATCAGAAGC
p38	CGATGAGTTTGCAGCATCC
p39	CTACCCGTAACCTTACAG
p40	TGGAAGAAATGACACGAG
P41	GCAGGCTGAAACAGCATTG
P42	GTTCCGCGAATCATAAGCC
P43	GCACAGTCGTTGTACAAATTCGTATTCCC
P44	ACGATTCTAAACGCCTCTTGTTACGATC

Supplementary Figure S1

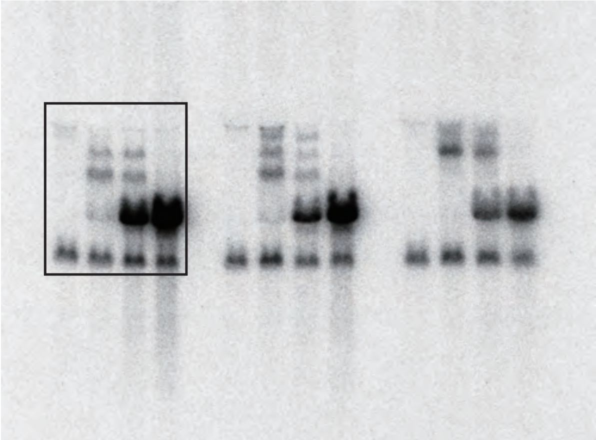

Uncropped blot used in Figure 1A

Supplementary Figure S2

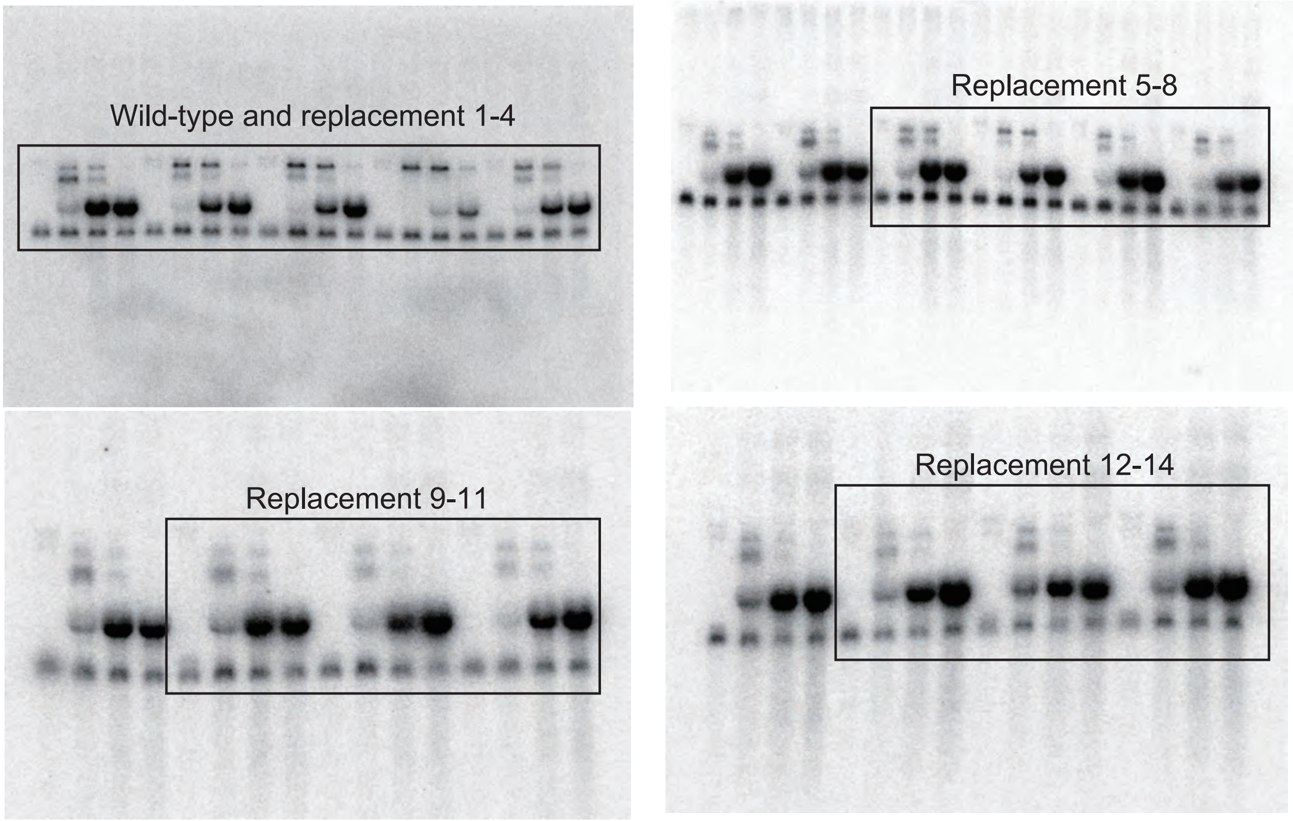

Uncropped blot used in Figure 1C

Supplementary Figure S3

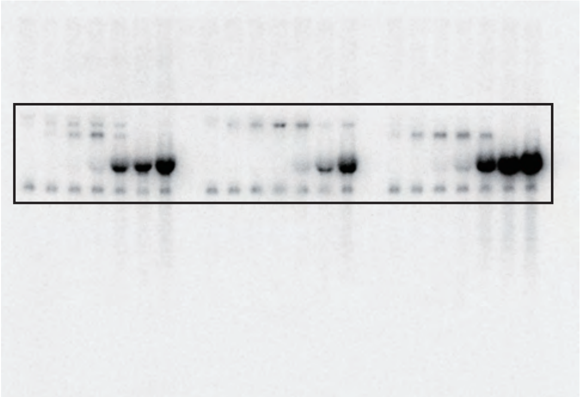

Uncropped blot used in Figure 2

Supplementary Figure S4

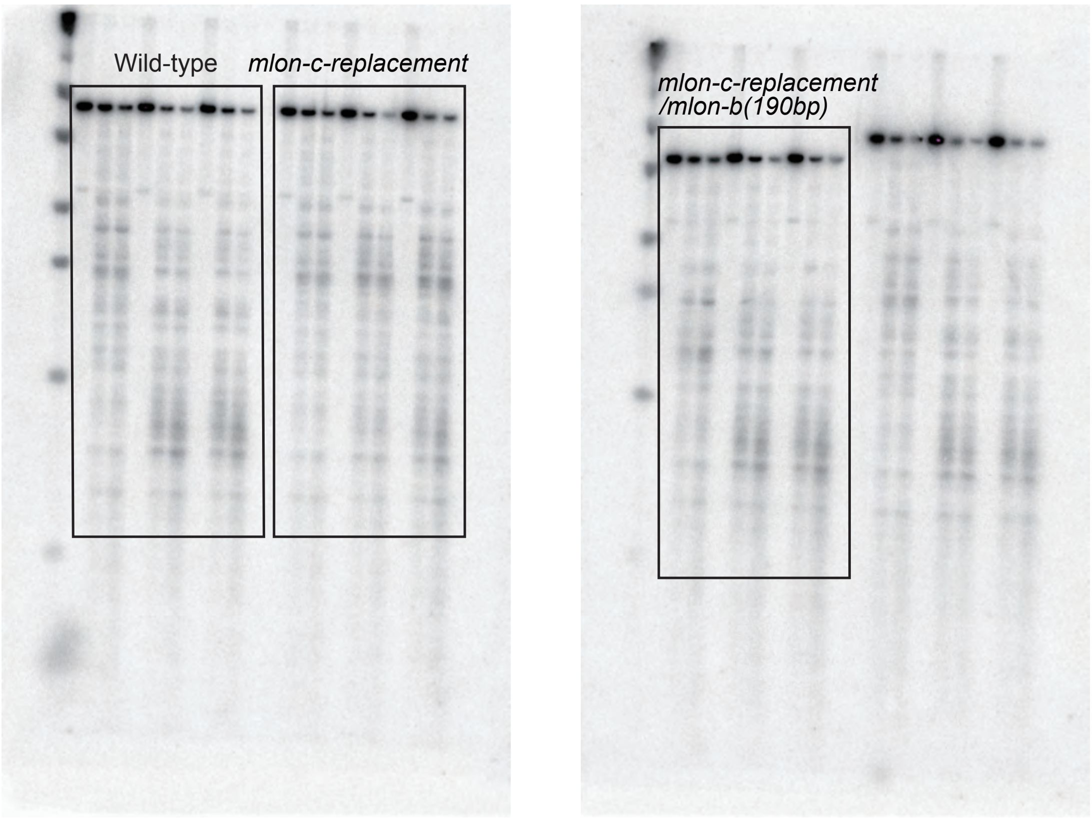

Uncropped blot used in Figure 3A

Supplementary Figure S5

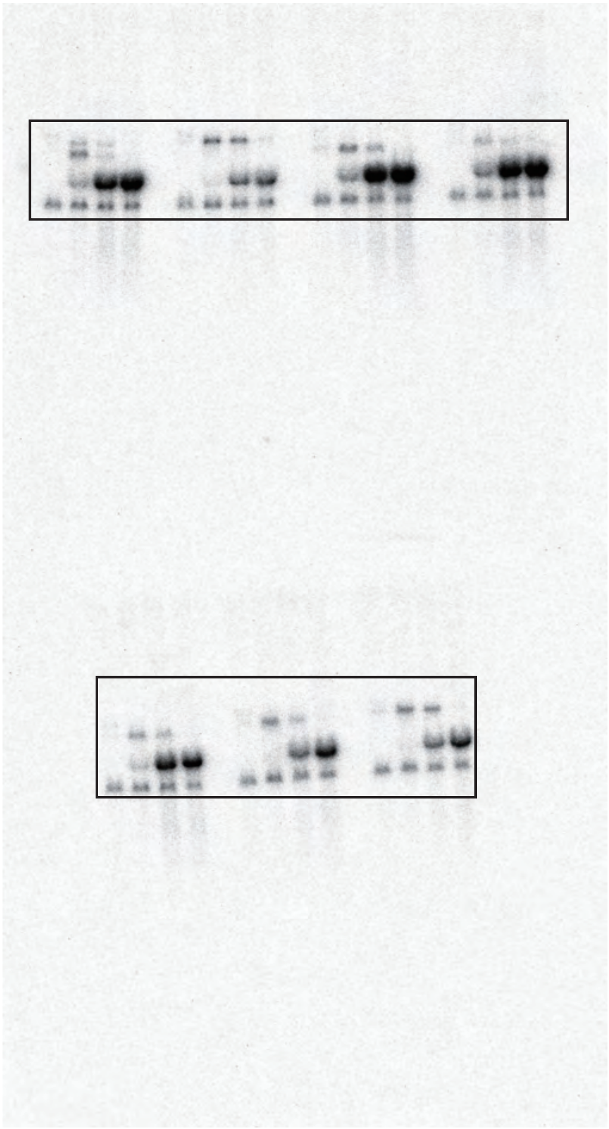

Uncropped blot used in Figure 4
